# Review of Contagious Abortion1Read before the Students’ Society of the McKillip Veterinary College.

**Published:** 1900-03

**Authors:** S. P. Smith

**Affiliations:** Student, Chicago, Ill.


### REVIEW OF CONTAGIOUS ABORTION.[1]

By S. P. Smith,
STUDENT, CHICAGO, ILL.

Cases of animals affected with this disease were recorded as early as the third century B.C., and since that date it has become slowly disseminated, and to-day it exists quite extensively throughout the civilized continents. In our State of New York alone this malady is given the credit of being the etiology of an annual loss of several million dollars worth of livestock and dairy products.

The aggregated statistics and observations of the past show that the bovine species is the most susceptible and the one most often to contract this disease, though it is by no means rare in the equine and ovine species, and occasionally found existing in the canine and feline families. Our present knowledge of the etiology and pathology of the disease is indicative of its being analogous to that of contagious abortion existing in the human family, and no doubt scientists in the near future will clearly demonstrate this valuable point, thus adding one more infectious disease to the list of contagious diseases that are in common with the human and with the domestic animals.

Prior to 1895 varied and extensive experiments were performed which demonstrated and confirmed its infectious and transmissible properties. By introducing vaginal exudates and fœtal membranes

[1] Read before the Students' Society of the McKillip Veterinary College.

of infected animals into the vagina of healthy, pregnant animals with few exceptions produces abortion in due time.

In view of its infectious nature the French Minister of Agriculture in 1885 authorized M. Nocard to make pathological and bacteriological researches to discover its exact etiology. After spending much time in careful laboratory work on the tissues of the uterus and fœtus of infected animals, he concluded that it was due to a specific pathogenic microörganism localized in the fœtus and its adnexas, though the two germs which he isolated from between the uterus and its embryo—one a micrococcus, the other a bacillus—proved not to be the specific organism of the disease ; but his labor was not all in vain, as he very accurately described the clinical symptoms and pathological anatomy of the disease. His expended energies opened the way and stimulated other scientists to make further bacteriological researches.

Ten years later Bang and Stribalt, of Copenhagen, took up the work anew. They selected for their material to work upon a cow from an infected herd, seven months in gestation and manifesting all the clinical symptoms of the disease. An autopsy was made, and the uterus containing the fœtus was removed to the laboratory. Upon microscopical examination of the straw-colored exudate in the uterus, large numbers of small, isolated, immobile bacilli were revealed. By isolating and experimenting with this bacillus, it proved to be the specific etiology of contagious abortion. These bacilli are about the same length as the bacillus malli, but are of an oval shape, resembling the hog-cholera bacilli, except being very much smaller, requiring a high-power objective to detect them ; they contain from three to four granules, which take a deeper stain than the rest of the germ, presenting an unevenly stained appearance. They stain best with aniline, as the common stains do not penetrate. They require a special culture media, though making a faint growth in blood serum and glycerin-agar. In these media the germs become diffused through the media, giving them a cloudy appearance, and by the fifth day this growth granulates, forming a deposit in the bottom of the tube. Stribalt's special serum gelatin-agar media is most favorable for proliferation. In three days' time this media forms a zone in the tube 5 mm. below the surface of the media, and 12 mm. in thickness, thus showing that it is neither an aërobic nor an anaërobic, but occupying an intermediate position, requiring 20 per cent. of the normal amount of oxygen supplied by the atmospheric air. In this respect it differs from all other known microörganisms.

Its pathological effect on laboratory animals is very marked. Pregnant guinea-pigs treated subcutaneously with 1 c.c. of a four-day culture of the bacilli causes them to abort in three to five days after injection. Rabbits thus treated with cultures of the germ show all the clinical and post-mortem symptoms of rabbits infected with the hog-cholera bacilli, producing death in about 75 per cent. This bacilli belongs to the same class as the hog-cholera bacilli, and they resemble each other very closely in their morphology and biological characteristics. Cultures of the bacilli may be subjected to a freezing temperature for seven months and retain all of their pathogenic properties. Pure virulent cultures have been isolated from a mummified fœtus retained in the uterus nine months after death.

Pregnant cows receiving pure cultures of the germ in the vagina give premature birth to their fœtus in about ten weeks after receiving the injection, but the period of incubation has a wide variation, and the time corresponds directly with the susceptibility and stage of gestation of the animal, though the primipara succumb most readily to its effects. Some infected females will carry their fœtus to full term, giving birth to a small, inferior offspring, which usually dies in a short time after birth from diarrhœa and enteritis, in which pathological process the specific bacilli are involved.

On an infected farm it may coexist in the pregnant females of the different species present, and can be readily transferred from one species to another by the use of the infected vaginal exudates. Ewes in the period of gestation treated hypodermatically with 18 c.c. of a pure culture produces pyrexia, dyspnœa, anorexia, and about the twelfth day abortion. Intravenous inoculation into pregnant mares causes them to abort on an average of about the twenty-eighth day.

The bacilli with some difficulty can be isolated from the fœtus and uterine discharge of all abortions caused by infection with this specific microörganisms.

The pathological anatomy is principally confined to the generative organs. The serous membranes surrounding the uterus and organism is normal, and the os uteri is hermetically sealed before parturition. There is catarrh of the uterine walls, accompanied with an abundant straw-colored, odorless, viscid, mucous exudate, of an alkaline reaction, between the mucous membrane of the uterus and chorion. This, in circumscribed areas, is of a gelatinous consistency. Between the chorion and alantoid exists a clear, gelatinous fluid, mixed with membranous particles. The alantoid fluid holds in suspension small clots, while the amniotic fluid is normal. The umbilical cord of the fœtus is œdematous, accompanied with congestion of the intestines and ascites.

The germ is present in all of these pathological lesions and in the blood of the fœtus. Therefore, we can consider epizoötic abortion a specific uterine catarrh, due to a specific microörganism. The uterine discharges accompanying this disease being odorless, differentiates it from metritis.

Males become infected by serving infected females, causing a specific urethritis, which is of a chronic nature and presents no external symptoms.

The pathogenesis of this disease has not been closely investigated, though it is generally accepted that the most common way it gains the animal economy is through the medium of the vagina from the infected floors, walls, and litter of stables, tails of infected animals in adjoining stalls, and fences, trees, forage with which infected animals have come in contact may also be the source of infection. The most active agent in disseminating the disease is the males used for breeding large numbers of females, especially females coming from different herds. The vagina is infected by the male with the bacilli at the time of sexual intercourse, and the organisms may pass through the os uteri with the spermatozoa; but when infection takes place through the vagina of pregnant animals it must necessarily be transferred to the uterus through the medium of the circulatory system, becoming localized in the uterus and its contents; this being the most favorable media in the animal body, and the imperfect circulation of these parts, favors its localization. The dairy herds are most prone to the disease, and perhaps due to the impaired vitality of the system and the development of the lacteal organs, through generations of breeding, to the expense of the generative organs. As yet nothing is known of infection taking place through the respiratory and alimentary tracts, though it may readily gain the circulation through abrasions of the skin.

The clinical symptoms resemble very closely those of normal parturition, except the tumefaction of the vulva and udder takes place in a much shorter time, and is accompanied by a vaginal discharge. The majority of abortions take place about the seventh month of gestation, though it may occur at any stage of gestation. The fœtus is usually alive at the time of parturition, but dies in a short time after birth. Cows once infected carry their fœtuses a few weeks later in the period of gestation each succeeding year, until the third or fourth year they have acquired immunity and still may remain as a source of infection to other males and females.

The prophylactic treatment is of most importance in combating this disease: strict isolation of infected animals, followed by antiseptic irrigations to the generative organs, and their former habitat thoroughly cleansed and disinfected. Exposed females have been carried to the point of normal parturition by repeated hypodermatic injections of a weak carbolic acid solution. The more recent French treatment promises better results and consists of injections into the muscular tissue every third or fourth day with 10 c.c. of a solution composed of one part turpenol and nine parts olive oil. This treatment is begun in suspected cases about the seventh month of gestation. Another prophylactic agent which has yet to prove its efficiency by experimentation is the antitoxin of contagious abortion made from pure cultures of the bacilli by attenuating their virulence with heat.

